# Age-related and sex-specific trends in sleep quality in children and adolescents

**DOI:** 10.3389/fnins.2025.1581929

**Published:** 2025-07-30

**Authors:** Hugi Hilmisson, Solveig Dora Magnusdottir, Robert Joseph Thomas

**Affiliations:** ^1^MyCardio LLC, SleepImage^®^, Denver, CO, United States; ^2^Division of Pulmonary, Critical Care and Sleep Medicine, Department of Medicine, Beth Israel Deaconess Medical Center, Boston, MA, United States

**Keywords:** children, adolescents, sleep quality, sleep quality index, sex differences, sleep trends

## Abstract

**Introduction:**

Strong developmental trends are well described in non-rapid eye movement (NREM) sleep characteristics but also seen in cyclic-alternating-pattern (CAP). The latter shows a bimodal distribution: slow wave dominant (A1) complexes early in life and A2/A3 complexes later in life. This analysis aimed to assess trends in CAP-linked cardiopulmonary coupling (CPC) calculated Sleep Quality Index (SQI) from childhood through adolescence.

**Methods:**

Analysis of de-identified data from the SleepImage^®^ System (MyCardio LLC, Denver, CO, United States), using CPC-calculations evaluating integrated electrocortical-autonomic-respiratory interactions to derive sleep states, SQI, and combined with oxygen saturation, an apnea hypopnea index (AHI).

**Results:**

Forty-one thousand nights of continuous sleep recordings of ≥ 6 h in duration and ≥ 4 h of total sleep time (TST), with good signal quality (≥ 80%) from individuals < 18 years of age were included in the analysis (48% girls-52% boys). Age groups were defined as 2–5 years (preschool-age, 39% girls-61% boys), 6–9 years (school-age, 47% girls-53% boys), 10–13 years (early-adolescent, 47% girls-53% boys), 14–17 years (late-adolescent, 52% girls-48% boys). In the cohort 20% had moderate- (AHI_3%_ 5–10) and 8% severe sleep apnea (AHI_3%_ ≥ 10). SQI is highest in school-aged children that are expected to sleep for 9–12/24 h with no sex differences observed (75.8 ± 15.8 and 75.3 ± 16.2; *p* = 0.06). Preschool-aged children are expected to sleep for 10–13/24 h, have a slightly lower SQI compared to school-aged children, with SQI higher in girls (73.4 ± 17.5 and 71.6 ± 19.2; *p* < 0.001). During early adolescence, when sleep duration is expected to be 8–10/24 h, SQI is significantly lower in girls compared to boys (70.5 ± 17.4 and 71.8 ± 17.0; *p* < 0.001). In late adolescence, SQI decline continues, but at a slower rate in girls who, at this age, girls have higher SQI than boys (63.1 ± 18.3 and 60.5 ± 18.2); *p* < 0:001. AHI_3%_ is significantly lower in girls in all age-groups; it is lowest in school-age children and gradually increases during adolescence.

**Conclusion:**

Children seem to reach their full potential in sleep stability and quality around school-age. In early adolescence, measured sleep stability and quality start to gradually decline, with the decline starting earlier in girls while larger in boys during the adolescent years.

## Graphical Abstract

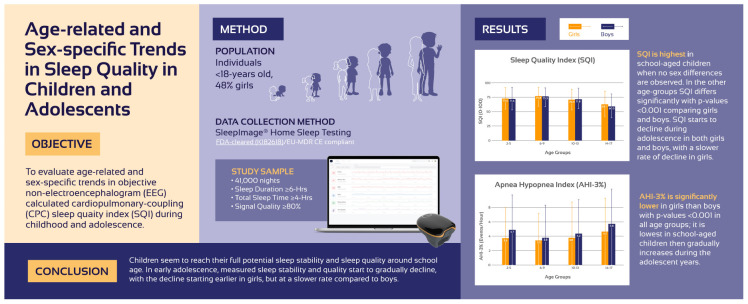

## 1 Introduction

Many physiological functions, including sleep duration and sleep patterns, are associated with age-related changes during childhood and adolescence. With progressing age sleep duration declines, with recommended sleep duration (per 24 h) for preschool-aged children 10–13 h, school-aged children 9–12 h and adolescents is 8–10 h (Paruthi et al., 2016). Sleep patterns also change with polysomnography (PSG) recorded amount and amplitude of non-rapid eye movement (NREM) showing slow wave sleep (SWS; 1–4 Hz delta EEG) decreasing, and the duration of NREM stage-1 and stage-2 increasing (Scholle et al., 2011). Cyclic alternating patterns (CAP) is a measure of EEG-estimated NREM-sleep stability, which increases during childhood, peaks at puberty, and then decreases during adolescence (Parrino et al., 2012; Parrino et al., 2014). After adolescence, an increase in CAP continues with advancing age. The early increases are dominated by slow-wave phasic complexes (A1 CAP), while later-life CAP shows substantial dominance of faster EEG activities (A2/A3 CAP).

Restorative and regenerative functions of sleep depend on age and adaptation to demands. In healthy children the rapid advances in growth, cognition and behavior are reflected in sleep duration and sleep architecture, but sex differences in sleep may be more difficult to capture (Lokhandwala and Spencer, 2022; Mason et al., 2021). During adolescence, when children reach puberty, sexual hormones and their related changes begin to affect sleep architecture and sex differences in sleep quality become more evident (Pengo et al., 2018). After puberty, the anatomy of the upper airway and its collapsibility, arousal response and ventilatory control changes which can influence sex differences in prevalence of sleep disordered breathing (SDB) (Lozo et al., 2017), which may impact sleep quality.

Studies comparing sex differences in sleep quality derived from EEG based PSG sleep studies (Barbato, 2021) have found that females often report lower subjective sleep quality than males, despite having better sleep quality when objectively evaluated with PSG (Markovic et al., 2020; Mong and Cusmano, 2016).

Non-EEG methods have been developed to assess sleep quality, including analysis of movement, peripheral arterial tone, and autonomic activity in respiratory and cardiovascular interactions. Cardiopulmonary-coupling (CPC) analysis provides an integrated output of electrocortical modulation of cardiovascular and respiratory-autonomic interactions, a foundation for sleep quality evaluation. An embodiment of this technology is the SleepImage^®^ System (United States Food and Drug Administration, (US FDA-cleared) and European Union Medical Device Regulatory (EU-MDR CE-marked) compliant). The input signals are heart rate variability (HRV) and respiratory tidal volume variability (TVV), and the output including sleep stability measures and the Sleep Quality Index (SQI) which is heavily weighted by stable NREM sleep [high frequency coupling (HFC)]. Combining the CPC-output with oxygenation information (SpO_2_), a PSG-equivalent FDA cleared apnea hypopnea index (AHI) is generated (Al Ashry et al., 2021a,b; Lu et al., 2023; Magnusdottir et al., 2020; Thomas et al., 2005; Thomas et al., 2014; Wood et al., 2020). In children, the SQI has demonstrated relationship with cardiometabolic health (Hilmisson et al., 2019; Magnusdottir et al., 2022), cognition and behavior (Magnusdottir et al., 2021), memory and learning (Yuanjie et al., 2025).

The aim of this study was to estimate developmental dynamics of sleep quality/stability, age and sex related trends across childhood and adolescence, based on the CPC-calculated SQI.

## 2 Materials and methods

This retrospective analysis of de-identified data analyzed by the SleepImage System (MyCardio LLC, Denver, CO, United States; SleepImage), a Health Insurance Portability and Accountability Act (HIPAA) compatible Software as a Medical System (SaMD) that is US FDA-cleared (K182618) and EU-MDR CE compliant. The SaMD can analyze plethysmography (PLETH) and SpO_2_ data based on data acquisition characteristics from approved devices. The data in this analysis was collected with the SleepImage Ring (SR), that includes a photoplethysmography-sensor (PPG) that collects continuous PLETH-signal and SpO_2_-data. The SR connects over Bluetooth to the SleepImage Mobile Application, a non-Medical Device Data System (MDDS) that stores the data during the sleep recording; and at the end of recording the data is transferred to the SleepImage-SaMD cloud from the MDDS for automatic analysis.

Data including age, gender and sleep output was extracted from the SleepImage System, a HIPAA compliant database, to analyze age and sex related trends in sleep quality comparing girls and boys, based on the FDA-cleared and proprietary Sleep Quality Index (SQI). On average there were 3-nights of sleep recordings for each participant in the analysis. Informed consent for this analysis was not required as the data extracted was de-identified, permitting use under HIPAA and CCPA; for further information, please refer to the SleepImage Privacy Policy. No clinical data was accessible.

### 2.1 Cardiopulmonary coupling

The CPC-method applies mathematical methodologies to generate the output, based on physiological measures calculating heart (HRV) or pulse (PRV) rate variability (R-R interval time series) and fluctuations in R-wave/pulse-wave amplitude induced by respiration to detect changes in breathing (TVV, tidal volume variability). These outputs are strongly modulated by sleep-wake state and stages. The cross spectral power and coherence of the RR-time series and corresponding TVV-time series are calculated for consecutive windows and a product of coherence and cross-spectral power is used to obtain the ratio of coherent cross power in the low frequency [Low frequency Coupling (LFC), 0.01–0.1 Hz] to that in the high-frequency band [High Frequency Coupling (HFC), 0.1–0.4 Hz]. The logarithm of the high to low frequency CPC-ratio is then computed to yield a continuously and moving average of overlapping CPC windows and output of stable-NREM sleep and unstable-NREM sleep, REM-sleep and wake. Graphing CPC at relevant frequencies (ordinate) vs. time (abscissa) provides the SleepImage spectrogram (Al Ashry et al., 2021b; Thomas et al., 2005; Hilmisson et al., 2020).

Stable NREM-sleep relates to a global condition of brain oscillation stability when all the subsystems that control and influence the sleep mechanisms are in balance/harmony. Stable NREM-sleep is characterized by stable breathing and stable oxygenation, high vagal tone, non-cyclic alternating pattern (n-CAP) on the electroencephalogram (EEG) (Parrino et al., 2012), continuous occurrence of slow oscillations, high delta power, blood pressure dipping and stable arousal threshold. This state could be considered as “effective” (performing core functions) NREM-sleep. Effective sleep enables the desirable functions of sleep across multiple dimensions (e.g., neuronal networks, cardiovascular, metabolic, immune etc.) such that spending periods in this state enables recovery and restorative processes.

Unstable NREM-sleep is a marker of sleep instability that has exactly the opposite features of Stable NREM-sleep, with variability in TVV, cyclic variation in heart rate (CVHR), CAP on EEG, low relative delta power, non-dipping of blood-pressure and unstable arousal thresholds. This state may be considered as “ineffective” NREM-sleep. Two pathological patterns are calculated during Unstable sleep; (1) elevated low frequency coupling broad-band (e-LFC_BB_), an indicator of sleep pathology such as pain, insomnia, anxiety and/or disordered breathing patterns like Obstructive Sleep Apnea (OSA) and Upper Airway Resistance Syndrome (UARS) and (2) elevated low frequency coupling narrow-band (e-LFC_NB_) identifying a periodic-type breathing and heart-rate patterns indicating sustained periods of periodic breathing and central sleep apnea (CSA) or “physiologic” periodicity due to Periodic Limb Movements in Sleep (PLMS) when drop in SpO_2_ is not observed (Al Ashry et al., 2021b; Thomas et al., 2007; Thomas et al., 2005; Thomas et al., 2014; Wood et al., 2020). The Sleep Quality Index (SQI) is a proprietary summary index of the CPC biomarkers of sleep quality, sleep stability, fragmentation, and periodicity, which provides a meaningful unit of measure of sleep health. The SQI is displayed on a scale of 0–100 with expected values for both children and adults (Hilmisson et al., 2019; Magnusdottir et al., 2020; Magnusdottir et al., 2022; Yuanjie et al., 2025).

### 2.2 Outcome measures

The primary outcome measures were to evaluate age-related and sex-specific trends in SQI during childhood and adolescence, based on pre-defined age-groups.

### 2.3 Statistical analysis

Descriptive statistics are presented as means with standard deviation (± SD). Analysis of variance (ANOVA) was utilized to investigate different categorical variables (groups) including age and genders on dependent variables measuring sleep quality, sleep stability, sleep fragmentation and sleep apnea. The calculations were based on a simple average, not a weighted average. The Shapiro-Wilk test was used to test for normality, which revealed that all dependent variables for which results are presented are normally distributed. Levene’s test was used to assess homogeneity of variances.

Analysis of variance was chosen as the statistical method due to its effectiveness in assessing whether the means of two or more groups are significantly different from each other. ANOVA was chosen in favor of multiple *t*-tests for its reduced risk of type I error when comparing differences among group means. *Post hoc* analysis was performed using the Games-Howell *post hoc* method for pairwise comparisons to identify differences between the groups.

Pearson’s correlation (r) analysis was utilized to assess associations between two quantitative variables, separately for each sex. For all statistical analysis *p* < 0.01 was considered significant.

SciPy version 1.10.1^1^ and Pingouin version 0.5.5^2^ were used for the analysis.

## 3 Results

### 3.1 Sleep recordings

Forty-one thousand nights of sleep recordings of ≥ 6 h in duration and ≥ 4 h of total sleep time (TST), with good signal quality (≥ 80%) from individuals < 18 years of age were included in the analysis (48% girls). Age groups were defined as 2–5 years (*n* = 3,738; 39% girls/61% boys, average three-nights), 6–9 years (*n* = 14,025; 47% girls/53% boys, average three-nights), 10–13 years (*n* = 12,430; 47% girls/53% boys, average three-nights), 14–17 years (*n* = 10,087; 52% girls/48% boys, average four-nights).

The dataset that was expected to be clinically enriched and skewed toward children evaluated for sleep disordered breathing (Table 1); 37% did not have sleep apnea (AHI_3%_ < 2), 35% had mild sleep apnea (AHI_3%_ 2–5), 20% had moderate sleep apnea (AHI_3%_ 5–10) and 8% severe sleep apnea (AHI_3%_ ≥ 10). A prevalence marginally higher than could be expected in the general population (Magnusdottir and Hill, 2024).

**TABLE 1:** Comparison of sleep quality index (SQI), on pre-defined sleep apnea groups based on the apnea hypopnea index-3% (AHI_3%_; group-1 AHI_3%_ < 2, group-2 AHI_3%_ 2–5, group-3 AHI_3%_ 5–10, group-4 AHI_3%_ > 10) stratified based on pre-defined age groups (mean ± SD).

Age gropus	2-5 years; *n* = 3,738 G1 = 1,380 (36.8%) G2 = 1,351 (36.0%) G3 = 721 (19.2%) G4 = 301 (8.0%)	6-9 years; *n* = 14,025 G1 = 6,030 (42.8%) G2 = 5,042 (35.8%) G3 = 2,195 (15.6%) G4 = 813 (5.8%)	10-13 years; *n* = 12,430 G1 = 4,869 (39.0%) G2 = 4,218 (33.8%) G3 = 2,369 (19.2%) G4 = 996 (8.0%)	14–18 years; *n* = 10,807 G1 = 2,982 (27.5%) G2 = 3,595 (33.1%) G3 = 2,964 (27.3%) G4 = 1,309 (12.1%)
Group-1 AHI_3%_ < 2	81.5 (± 15.7)	81.8 (± 13.8)	78.4 (± 15.3)	70.8 (± 18.5)
Group-2 AHI_3%_ 2–5	71.8 (± 14.7)	73.5 (± 13.7)	70.8 (± 14.7)	64.8 (± 14.9)
Group-3 AHI_3%_ 5–10	62.5 (± 18.1)	67.1 (± 16.7)	62.1 (± 16.6)	54.4 (± 16.3)
Group-4 AHI_3%_ > 10	56.2 (± 23.1)	63.4 (± 21.8)	59.1 (± 20.2)	50.3 (± 17.7)
	*P*-values	*P*-values	*P*-values	*P*-values
Group-1 vs. Group-2	< 0.001	< 0.001	< 0.001	< 0.001
Group-1 vs. Group-3	< 0.001	< 0.001	< 0.001	< 0.001
Group-1 vs. Group-4	< 0.001	< 0.001	< 0.001	< 0.001
Group-2 vs. Group-3	< 0.001	< 0.001	< 0.001	< 0.001
Group-2 vs. Group-4	< 0.001	< 0.001	< 0.001	< 0.001

Group-1 37.1% (*n* = 15,261), Group-2 34.5% (*n* = 14,206), total with AHI < 5 71.6% (*n* = 29,467). SQI, sleep quality index; AHI, apnea hypopnea index; SD, standard deviation.

### 3.2 Sleep quality index

**FIGURE 1:**
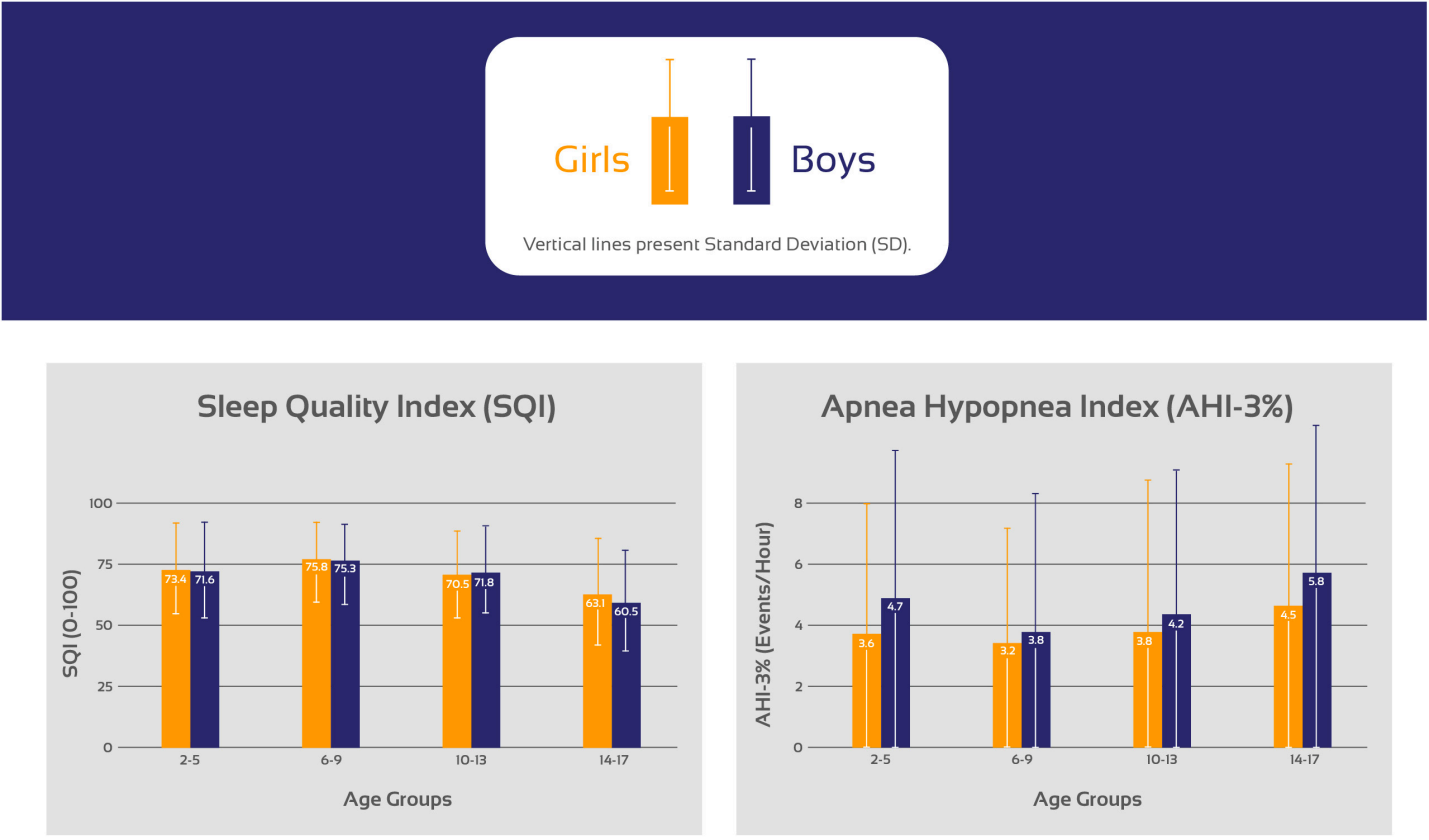
Trends in the sleep quality index (SQI) and the apnea hypopnea index (AHI) stratifying the cohort based on age in 3 years increments as preschool-aged children (2–5 years), school-aged children (6–9 years), early adolescents (10–13 years) and late adolescents (14–17 years).

Based on pre-defined age-groups, age-related and sex-specific sleep metrics comparing girls and boys based on pre-defined age-groups are presented in Figure 1 and Table 2. Included sleep parameters from the CPC-analysis are the sleep quality index (SQI), sleep efficiency (SE), sleep stability (HFC), sleep fragmentation (SF) and the apnea-hypopnea index (AHI_3%_). The SQI was highest in school-aged children (6–9 years) with no significant difference comparing girls (75.8) and boys (75.3), *p* = 0.06. Preschool-aged children had a lower SQI compared to school-aged children and preschool-aged girls have significantly higher SQI (73.4) than boys (71.6), *p* < 0.001. The SQI starts to decline in early adolescence, when girls have significantly lower SQI (70.5) compared to boys (71.8), < 0.001.

**TABLE 2:** Summary of results from analysis of 41,000 sleep studies (*n* = 41,000) aimed at evaluating if there are sex-specific differences in sleep quality, sleep stability and sleep disordered breathing in 2–18 years old children and adolesents (mean ± SD).

Age group	SQI			SE			Sleep stability			Sleep Fragmentation			AHI		
	Girls	Boys	*P*-value	Girls	Boys	*P*-value	Girls	Boys	*P*-value	Girls	Boys	*P*-value	Girls	Boys	*P*-value
A. 4–5 years *n* = 3,738 39% girls, 61% boys	73.4 (± 17.5)	71.6 (± 19.2)	< 0.001	84.1 (± 10.1)	81.9 (± 13.9)	< 0.001	60.2 (± 20.1)	56.9 (± 22.4)	< 0.001	6.6 (± 7.4)	6.6 (± 8.3)	0.978	3.6 (± 4.4)	4.7 (± 6.1)	< 0.001
B.6-9 years *n* = 14,025 47% girls, 53%boys	75.8 (± 15.8)	75.3 (± 16.3)	0.06	84.9 (± 9.6)	83.7 (± 10.3)	< 0.001	62.2 (± 18.4)	60.9 (± 18.9)	< 0.001	5.5 (± 6.6)	5.4 (± 7.0)	0.741	3.2 (± 4.0)	3.8 (± 4.5)	< 0.001
C.10-13 years *n* = 12430 47% girls, 53% boys	70.5 (± 17.4)	71.8 (± 17.0)	< 0.001	83.3 (± 9.9)	82.7 (± 10.5)	< 0.001	56.0 (± 19.9)	56.9 (± 19.4)	< 0.001	7.9 (± 8.9)	7.1 (± 8.0)	< 0.001	3.8 (± 4.7)	4.2 (± 4.8)	< 0.001
D.14-17 years *n* = 10807 52% girls, 48% boys	63.1 (± 18.3)	60.5 (± 18.2)	< 0.001	82.9 (± 9.5)	80.5 (± 11.0)	< 0.001	48.2 (± 20.3)	44.3 (± 20.2)	< 0.001	11.5 (± 10.9)	13.4 (± 11.4)	< 0.001	4.5 (± 4.7)	5.8 (± 5.7)	< 0.001

AHI, apnea hypopnea index; SE, sleep efficiency; SD, standard deviation; SQI; sleep quality index; sleep stability (high frequency coupling, HFC).

From early adolescence to late adolescence, the SQI decreased in both sexes while girls maintained higher SQI (63.1) compared to boys (60.5), *p* < 0.001. During childhood the sexes did not differ in SF but during early adolescence girls developed higher SF compared to boys that again changed during late adolescence when boys have higher SF compared to girls.

### 3.3 Correlation of CPC measures

The Pearson correlation coefficient (r) analysis is reported in Table 3. The SQI had the strongest negative correlation with SF, moderate negative correlation with SE, while the correlation with AHI_3%_ was low, in both sexes and in all age groups: The correlation of SQI with SF is strong and higher in girls compared to boys in all age groups −0.83 vs. −0.81 (2–5 years); −0.82 vs. −0.79 (6–9 years); −0.84 vs. −0.82 (10–13 years); −0.85 vs. −0.84 (14–17 years). The correlation of SQI with SE was moderate in young children and during early adolescence, lower in girls compared to boys −0.46 vs. −0.58 (2–5 years); −0.43 vs. −0.45 (6–9 years); −0.41 vs. −0.44 (10–13 years), changing to be stronger in girls compared to boys during late adolescence, but still moderate −0.43 vs. −0.40 (14–17 years). The correlation of SQI with AHI_3%_ is weak in all age groups; higher in girls compared to boys; −0.35 vs. −0.26 (2–5 years); −0.36 vs. −0.30 (6–9 years); −0.35 vs. −0.30 (10–13 years); −0.34 vs. −0.32 (14–17 years).

**TABLE 3:** Pearson correlation coefficient (r) evaluating correlation of the Sleep Quality Index (SQI) with the Apnea Hypopnea Index (AHI), sleep fragmentation (SF) and sleep efficiency (SF).

Age-groups								
	2–5 years		6–9 years		10–13 years		14–17 years	
	**AHI_3%_**		**AHI_3%_**		**AHI_3%_**		**AHI_3%_**	
	**Girls**	**Boys**	**Girls**	**Boys**	**Girls**	**Boys**	**Girls**	**Boys**
**SQI**	−0.35*	−0.26*	−0.36*	−0.30*	−0.35*	−0.30*	−0.34*	−0.32*
	**SF**		**SF**		**SF**		**SF**	
	**Girls**	**Boys**	**Girls**	**Boys**	**Girls**	**Boys**	**Girls**	**Boys**
**SQI**	−0.83***	−0.81***	−0.82***	−0.79***	−0.84***	−0.82***	−0.85***	−0.84***
	**SE**		**SE**		**SE**		**SE**	
	**Girls**	**Boys**	**Girls**	**Boys**	**Girls**	**Boys**	**Girls**	**Boys**
**SQI**	0.46**	0.58**	0.43**	0.45**	0.41**	0.44**	0.43**	0.40**

All *p*-values for the comparisons are < 0.0001 most likely due to the large samples size and therefore not shown in the table. Magnitude/strength of correlation is marked as:

*** high correlation (0.70–0.89),

**Moderate correlation (0.40–0.69),

*low/week correlation (0.10–0.39) (Schober et al., 2018).

### 3.4 Sex differences

Table 4 shows that school-aged girls have significantly higher SQI, better SE, less SF and lower AHI_3%_ than preschool-aged girls (Figure 1). During the adolescence years there was a negative trend with a decrease in SQI, SE as SF and AHI_3%_ increases. Preschool-aged children had on average significantly higher AHI_3%_ than school-aged children and during the adolescence years there was a gradual significant increase in AHI_3%_ with age. Table 5 shows that school-aged boys had significantly higher SQI, better SE, less SF and lower AHI_3%_ than preschool-aged boys (Figure 1). In boys there are not statistically significant differences between preschoolers and early adolescents in SQI, SE, sleep stability, sleep fragmentation or AHI_3%_. Preschool-aged boys had on average higher AHI_3%_ than school-aged boys, and during the adolescence years there was a gradual significant increase in AHI_3%_ with age.

**TABLE 4:** Evaluation of sleep quality, stability, fragmentation and disordered breathing in girls (47.6% of cohort) based on predefined age groups.

	SQI		SE		Sleep stability		Fragmentation		AHI	
Age group	Mean (± SD)		Mean (± SD)		Mean (± SD)		Mean (± SD)		Mean (± SD)	
A. 2–5 years (*n* = 1,458)	73.4 (± 17.5)		84.1 (± 10.1)		60.2 (± 20.1)		6.6 (± 7.4)		3.6 (± 4.4)	
B. 6–9 years (*n* = 6,592)	75.8 (± 15.8)		84.9 (± 9.6)		62.2 (± 18.4)		5.5 (± 6.6)		3.2 (± 4.0)	
C. 10–13 years (*n* = 5,842)	70.5 (± 17.4)		83.3 (± 9.9)		56.0 (± 19.9)		7.9 (± 8.9)		3.8 (± 4.7)	
D. 14–17 years (*n* = 5,620)	63.1 (± 18.3)		82.9 (± 9.5)		48.2 (± 20.3)		11.5 (± 10.9)		4.5 (± 4.7)	
**One-way ANOVA (F,p)**										
	**Difference**	***P*-value**	**Difference**	***P*-value**	**Difference**	***P*-value**	**Difference**	***P*-value**	**Difference**	***P*-value**
A versus B	−2.4	< 0.001	−0.8	0.203	−2.0	0.02	1.1	< 0.001	0.4	< 0.001
A versus C	2.9	< 0.001	0.8	0.153	4.2	< 0.001	−1.3	< 0.001	−0.2	< 0.001
A versus D	10.3	< 0.001	1.2	0.001	12.0	< 0.001	−4.9	< 0.001	−0.9	< 0.001
B versus C	5.3	< 0.001	1.6	< 0.001	6.2	< 0.001	−2.4	< 0.001	−0.6	< 0.001
B versus D	12.7	< 0.001	2.0	< 0.001	14.0	< 0.001	−3.0	< 0.001	−1.3	< 0.001
C versus D	7.4	< 0.001	0.4	0.429	7.8	< 0.001	−3.6	< 0.001	−0.7	< 0.001

AHI, Apnea Hypopnea Index; Fragmentation, elevated low-frequency coupling broadband, eLFC_BB_; SD, standard deviation; SE; sleep efficiency; sleep stability (high frequency coupling, HFC), SQI, sleep quality index.

**TABLE 5:** Evaluation of sleep quality, stability, fragmentation and disordered breathing in boys (52.4% of cohort) based on predefined age groups.

	SQI		SE		Sleep stability		Fragmentation		AHI	
Age group	Mean (± SD)		Mean (± SD)		Mean (± SD)		Mean (± SD)		Mean (± SD)	
A. 2–5 years (*n* = 2,280)	71.6 (± 19.2)		81.9 (± 13.9)		56.9 (± 22.4)		6.6 (± 8.3)		4.7 (± 6.1)	
B. 6–9 years (*n* = 7,433)	75.3 (± 16.3)		83.7 (± 10.3)		60.9 (± 18.9)		5.4 (± 7.0)		3.8 (± 4.5)	
C. 10–13 years (*n* = 6,588)	71.8 (± 17.0)		82.7 (± 10.5)		56.9 (± 19.4)		7.1 (± 8.0)		4.2 (± 4.8)	
D. 14–17 years (*n* = 5187)	60.5 (± 18.2)		80.5 (± 11.0)		44.3 (± 20.2)		13.4 (± 11.4)		5.8 (± 5.7)	
**One-way ANOVA (F,p)**										
	**Difference**	***P*-value**	**Difference**	***P*-value**	**Difference**	***P*-value**	**Difference**	***P*-value**	**Difference**	***P*-value**
A versus B	−3.7	< 0.001	−1.8	< 0.001	−4.0	< 0.001	1.2	< 0.001	0.9	< 0.001
A versus C	−0.2	0.999	−0.8	0.359	0.0	1.000	−0.5	0.416	0.5	0.07
A versus D	11.1	< 0.001	1.4	< 0.001	12.6	< 0.001	−6.8	< 0.001	−1.1	< 0.001
B versus C	3.5	< 0.001	1.0	< 0.001	4.0	< 0.001	−1.7	< 0.001	−0.4	< 0.001
B verus D	14.8	< 0.001	3.2	< 0.001	16.6	< 0.001	−8.0	< 0.001	−2.0	< 0.001
C versus D	11.3	< 0.001	2.2	< 0.001	15.6	< 0.001	−6.3	< 0.001	−1.6	< 0.001

AHI, apnea hypopnea index; Fragmentation, elevated low-frequency coupling broadband, eLFC_BB_; SD, standard deviation; SE, sleep efficiency; sleep stability (high frequency coupling, HFC); SQI, sleep quality index.

Girls have significantly lower AHI_3%_ in all age groups compared to boys. Week correlation of AHI_3%_ with SQI was observed and stronger in girls than boys.

### 3.5 Age-related trends in sleep Quality related to sleep apnea severity

The pathology of sleep apnea includes both the severity and frequency of oxygen-desaturations and sleep fragmentation during sleep. Table 1 shows how increase in sleep apnea severity negatively affects sleep quality in all the predefined age-groups in both girls and boys.

## 4 Discussion

This analysis evaluated sleep quality in children and adolescents using the CPC-method and whether age-related and/or sex-specific trends were observed during childhood and adolescence. Age and sex differences were noted, as described above. The absolute differences were small.

Though all the studies were performed clinically, presumably for concern of sleep apnea, the results give a reasonable view of sleep stability/quality dynamics across the growth and development years, with 72% of the cohort having no- or mild sleep apnea and when apnea is known to have only mild effects on PSG sleep architecture. Children with significant comorbidities would also likely undergo laboratory polysomnography, so we presume this is a relatively healthy group of children and adolescents. It would be difficult to collect data on trends in sleep metrics in thousands of healthy children, though that would be ideal.

Children reach their full potential SQI and sleep stability around school-age which may be linked to age-related development in maturation and integration of subcortical and cortical subsystems of sleep, which are necessary to generate sleep stability (HFC). During early adolescence, SQI and sleep stability start to gradually decline. Brain development begins in prenatal life and is accompanied by dramatic changes in brain gray and white matter that increases rapidly during early childhood and peaks around early adulthood (Alex et al., 2024; Anastasiades et al., 2022). Total brain white and gray matter volumes increase linearly with age and roughly at the same rate in girls and boys, though consistently on average larger in boys. The relative volume of thalamus has been found to be larger in girls during adolescence compared to boys. This may affect developmental changes in sleep stability and SQI during childhood and adolescence (Sussman et al., 2016). The SQI is heavily weighed by stable sleep (high frequency coupling, HFC), a metric correlated with SWS (Thomas et al., 2014). Even though slow waves can be generated at a cortical level, subcortical structures including thalamus have an active role in regulating expression of slow waves and coordinating with sleep spindles, a process that changes with maturation and age (Bergamo et al., 2024; David et al., 2013; Schreiner et al., 2022). The sex-based differences in the size of thalamus may possibly be related to the higher sleep stability and SQI seen in girls during adolescence compared to boys.

When comparing preschool-aged children to school-aged children there was an increase in stable sleep (3.2% girls, 6.5% boys). School-aged children had the highest SQI and sleep stability, which was not surprising given the rapid changes with both physical growth and brain development reflected in cognition and behavior in young children. During early adolescence years, a decline in sleep stability starts (girls 10%, boys 6.5%) and continues during late adolescence (girls 13.9%, boys 22.1%). Stable sleep is associated with occurrence of non-CAP on EEG (Thomas et al., 2005), that gradually increases from school-age, peaking during early adolescence and then starts to decline (Parrino et al., 2012). When sleep is evaluated during PSG-studies, a decrease in SWS has been reported to be approximately 14% between 5 and 15 years of age (Sun et al., 2023).

Sex hormones contribute to sexual dysmorphism in brain development (Goddings et al., 2014) and sex differences in sleep quality become more evident when children reach puberty, when sexual hormones begin to affect sleep architecture (Pengo et al., 2018), anatomy of the upper airway, airway collapsibility, arousal response to increased inspiratory resistance, and ventilatory control that explains the sex differences in SDB/OSA (Lozo et al., 2017). The AHI_3%_, the most common metric to evaluate SDB/OSA was lower in girls compared to boys in all age groups in this analysis. The AHI_3%_ was higher in preschool-aged children compared to school-aged children, similar results to what others have reported based on PSG-studies evaluating children for SDB/OSA (Bonuck et al., 2011; Fernandes et al., 2024). During the preschool-age, the primary cause for airway blocking causing SDB/OSA, after accounting for obesity and certain cranial structures such as midface deficiency and mandibular hypoplasia that may affect the size of the upper airway, is often related to nasal resistance from adenoidal and/or tonsillar hypertrophy and that this tissue growth is often disproportionate to growth of the bony part of the nasopharyngeal space (Chuang et al., 2022). During school-age the AHI_3%_, is lower, but starts to increase again in early and late adolescence when the increase may be related to the hormonal changes occurring during puberty. The effect of progesterone are not fully understood but progesterone may enhance sleep quality and increase activity of the genioglossus muscle dilating the upper airway, decreasing upper airway resistance with positive impact on breathing, lower SDB/OSA and better sleep quality during the luteal phase compared to the follicular phase in girls (Haufe et al., 2022).

How may the type of information from this study be used? Charts reflecting developmental components are standard in pediatric practice, including weight/height (growth), language, and behavior. The sleep EEG shows well-known dynamic changes across development and sleep charts may add to evaluation of healthy growth and development. Though we were not able to track the same individual over time due to the nature of our data, various types of sleep assessments (e.g., questionnaires, activity trackers, EEG and autonomic-respiratory sleep analytics) will have distinct and clinically useable profiles in health and disease.

Limitations of this analysis include: (1) no data on medications and/or comorbid diseases was available; (2) no concomitant polysomnography given the nature of this data was available; and (3) no longitudinal data follow-up data in individual subjects was available. (4) Based on current guidelines that children who may suffer from sleep disorders should have a PSG-sleep study, it is not likely that children suffering from comorbid disorders were tested with a home sleep test. It is though possible that this dataset includes children with disorders that may impact sleep.

In conclusion, this analysis demonstrates that children seem to reach their full potential sleep quality, as estimated by cardiopulmonary coupling (CPC), around school-age. Starting from early adolescence, sleep stability and sleep quality start to gradually decline. SDB/OSA is higher in pre-school aged children compared to school-aged children and after early adolescence SDB/OSA starts to increase.

## Data Availability

The data analyzed in this study is subject to the following licenses/restrictions: the data collected during this study is not publicly available due to General Data Protection Regulations (GDPR) reasons. Requests to access these datasets should be directed to solveig.magnusdottir@sleepimage.com.

## Footnotes

1
https://scipy.org

2
https://pingouin-stats.org/build/html/index.html
